# Specific Depletion of Myelin-Reactive B Cells via BCR-Targeting

**Published:** 2015

**Authors:** A. V. Stepanov, A. A. Belogurov Jr., P. Kothapalli, O. G. Shamborant, V. D. Knorre, G. B. Telegin, A. A. Ovsepyan, N. A. Ponomarenko, S. M. Deyev, S. V. Kaveri, A. G. Gabibov

**Affiliations:** M.M. Shemyakin and Yu.A. Ovchinnikov Institute of Bioorganic Chemistry, Miklukho-Maklaya Str., 16/10, Russian Academy of Sciences, 117997, Moscow, Russia; Kazan Federal University, Kremlevskaya Str., 18, 420008, Kazan, Republic of Tatarstan, Russia; Institute of Gene Biology, Russian Academy of Sciences, Vavilova Str., 34/5, 119334, Moscow, Russia; Centre de Recherche des Cordeliers, Université Pierre et Marie Curie, UMR S 1138, F-75006, Paris, France

**Keywords:** multiple sclerosis, autoantigens, B cells, immunoglobulins, immunotoxins

## Abstract

B cells play a crucial role in the development and pathogenesis of systemic and organ-specific autoimmune diseases. Autoreactive B cells not only produce antibodies, but also secrete pro-inflammatory cytokines and present specific autoantigens to T cells. The treatment of autoimmune diseases via the elimination of the majority of B cells using the monoclonal anti-CD19/20 antibody (Rituximab) causes systemic side effects and, thus, requires a major revision. Therapeutic intervention directed towards selective elimination of pathogenic autoreactive B cells has the potential to become a universal approach to the treatment of various autoimmune abnormalities. Here, we developed a recombinant immunotoxin based on the immunodominant peptide of the myelin basic protein (MBP), fused to the antibody Fc domain. We showed that the obtained immunotoxin provides selective *in vivo *elimination of autoreactive B cells in mice with experimental autoimmune encephalomyelitis. The proposed conception may be further used for the development of new therapeutics for a targeted treatment of multiple sclerosis and other autoimmune disorders.

## INTRODUCTION

Multiple sclerosis (MS) is a chronic autoimmune neurodegenerative disease that affects the central nervous system, in which the major autoantigens are proteins of the myelin sheath of nerve fibers [1]. More than 200,000 people are affected by multiple sclerosis in the Russian Federation [2]. Despite the advances in the treatment of MS made in recent years, the existing therapeutics do not provide full recovery to the patient [3]. Modern approaches to the treatment of MS, including the introduction of recombinant antibodies and other low-molecular-weight agents specifically acting on components of the immune system, are prohibitively expensive for the budgets of developed countries and, taking into account the need for long-term care, endanger the entire rehabilitation system. Furthermore, the levels of disability of patients do not provide expectations for a positive prognosis in the social sphere. It is important to note that the current methods used in MS include primarily nonselective immunosuppressive drugs, which often cause systemic complications [4].

It has been considered for a long time that CD4^+^ T cells play a crucial role in the pathogenesis of MS. However now it is obvious that the B cell response undoubtedly plays an important role in the disease’s development. Autoreactive B cells not only produce autoantibodies, but they are also able to effectively function as antigen-presenting cells that in turn activate T cells [5]. In addition, B cells can secrete proinflammatory cytokines and enhance pathological self-destructive processes [6]. Therapy directed at a specific population of lymphocytes is, in the long term, a versatile remedy for a wide range of B cell disorders. Currently, elimination of autoreactive B cells is accomplished by administration of the monoclonal anti-CD19/20 antibody, Rituximab (Rituxan, MabThera), which is extensively used in the therapy of lymphomas and autoimmune diseases [7-12]. Clinical use of this drug is limited to a great extent and occurs in exceptional circumstances, since it leads to the elimination of most B cells in the body and, consequently, a wide range of side effects [13]. In this regard, the problem of developing drugs for specific therapy of multiple sclerosis and other autoimmune diseases remains rather topical.

The main approach to the treatment of multiple sclerosis is based on subcutaneous injection of an immunomodulating drug, glatiramer acetate (GA). GA is a polypeptide of 40–100 amino acid residues, comprising a random combination of alanine, lysine, glutamate, and tyrosine in a ratio of 4.5 : 3.6 : 1.5 : 1, respectively. The GA structure mimics one of the major autoantigens in MS, the highly positively charged myelin basic protein (MBP). The GA action presumably includes competition with fragments of the myelin basic protein for binding to MHC class II DR molecules, as well as induction of regulatory T cells (Th2/3 type) secreting anti-inflammatory IL-4 and IL-10 cytokines and the brain-derived neurotrophic factor [14]. It should be noted that the extent of GA therapy efficacy is highly fluctuant, up to full patient resistance to drug therapy [14].

In this paper, we suggest and successfully implement an approach to the development of recombinant polypeptides capable of specific depletion of abnormal lymphocytes *in vivo*. As a target for precise delivery of cytotoxic agents, we chose the surface immunoglobulin of autoreactive B cells (B cell receptor, BCR), which is a unique receptor that differentiates a certain, clonally homogeneous population of B cells from other cells of the organism. A number of studies have already demonstrated the high efficacy of targeted elimination of lymphocytes by BCR-specific immunotoxins *in vitro *[15, 16]. A high titer of autoantibodies specific for MBP has been previously shown in the serum of MS patients [17-19]. One of the most appropriate animal model of multiple sclerosis is experimental autoimmune encephalomyelitis (EAE) induced in SJL/J strain mice [20, 21]. Upon developing EAE in SJL/J mice, MBPspecific autoantibodies are also produced. Based on a previously generated library of recombinant MBP epitopes and using the enzyme-linked immunosorbent assay (ELISA), a comparative analysis of the specificity of the serum autoantibodies derived from MS patients and various EAE animals for different epitopes of the autoantigen was performed [22]. Based on the obtained data, the [QDENPVVHFFKNIVTPRTPPPSQ] MBP_82– 105_ immunodominant fragment was chosen as a highly specific ligand for surface BCRs of autoreactive B cells. Further, we developed a chimeric protein consisting of the MBP_82–105_ sequence fused to the antibody constant fragment that exhibits good pharmacodynamic parameters and, at the same time, can effectively induce the mechanisms of antibody-dependent cytotoxicity. In the present study, the therapeutic potential of the generated killer protein was studied in terms of selective depletion of autoreactive MBP-specific B cells *in vivo* in SJL/J strain mice with induced EAE.

## EXPERIMENTAL

**Development of the genetic construct encoding the immunoglobulin constant fragment fused with MBP_82–105_**

The nucleotide sequence encoding the MBP82-105 fragment was produced by PCR amplification with the mutually overlapping outer and inner primers 5’ATTAGGTACCCAAGATGAAAACCCCGTAGTCCACTTCTTCAAGA3’, 5’CGTAGTCCACTTCTTCAAGAACATTGTGACGCCTCGCACACC3’, and 5’TAATGTCGACTCCCTGCGACGGGGGTGGTGTGCGAGGCGTCACA3’. The PCR product was treated with the restriction endonucleases EcoRI and BgIII and then ligated with the similarly prepared pFUSE vector.

**Generation of clones producing recombinant molecules**

To generate a stable line of CHO cells producing the recombinant MBP_82–105_-Fc (pFUSE-MBP_82–105_-Fc) and Fc (pFUSE-Fc) molecules, CHO cells were transfected with appropriate genetic constructs. For this purpose, a day before transfection, CHO cells were split into wells of a six-well plate (Nunc) at a concentration of 0.5 million/mL. Upon reaching 80% confluency, the cells were transfected by lipofection using a Lipofectamine LTX kit (Invitrogen) according to the manufacturer’s recommendations. After 72 h, a medium with a selective antibiotic zeocin was added to the cells. The antibiotic- resistant cells were transferred to 96-well plates (Corning). The resulting clones were tested for the production of recombinant molecules by ELISA using monoclonal anti-Fc-antibodies.

**Isolation of Fc and MBP82–105-Fc molecules**

Isolation of killer proteins containing the Fc-fragment of a class IgG2a antibody was carried out as follows. Initially, the supernatant of CHO cells transfected with plasmids containing the nucleotide sequences encoding the antibody constant fragment fused with a peptide sequence was collected. The collected supernatant was centrifuged at 13,000 rpm for 10 min. After centrifugation supernatant was applied to an affinity chromatography column with the immobilized G protein (HiTrap Protein-G Sepharose, Amersham, USA) in PBS at a flow rate of 0.5 mL/min. The column was then washed with 80–100 PBS volumes at a speed of 2.5 mL/min to elute non-specifically adsorbed proteins. The fraction was eluted from the column with a 100 mM Glycine-HCl solution (pH 2.5) and immediately neutralized with a 2 M Tris base solution to pH 7.3–7.7. All chromatographic isolation stages were carried out on a DuoFlow BioRad system. Identification of Fc and MBP_82–105_-Fc samples and assessment of their purity were performed using denaturing polyacrylamide gel electrophoresis, followed by silver staining and an enzyme- linked immunoassay.

**Induction and therapy of EAE in SJL strain mice**.

The experiments were carried out at the Research and Production Department of the Branch of the Shemyakin- Ovchinnikov Institute of Bioorganic Chemistry, the Nursery for Laboratory Animals “Pushchino” (Russia, Pushchino), and at the Centre de Recherche des Cordeliers de Jussieu (CRC) (France, Paris) in compliance with all ethical standards. EAE was induced in SJL female mice at the age of 6 to 8 weeks with the SPF (specified pathogen free) status in accordance with the protocol [23] by a double injection of 100 μg of a mouse spinal cord homogenate (MSCH) in complete Freund’s adjuvant (CFA) containing tuberculin at a concentration of 4 mg/mL. On day 1, MSCH was injected subcutaneously into two points along the spinal column and, on the 3rd day, into the sole of hind feet. Additionally, on the day of MSCH in PAF injections, the mice were intravenously administered with a solution of the pertussis toxin (Calbiochem, USA) at a dose of 500 ng/mouse. Mice induced with EAE were divided into four groups of 10 animals, each: without injection (group without treatment); animals with a single injection of 200 μg of GA (Teva); animals in the groups Fc and MBP_82–05_-Fc were intravenously injected with 50 μg of the drug on days 5 and 10 after EAE induction. The severity of the autoimmune disease was evaluated on a daily basis according to the following scale: 0 –norm; 1 –loss of tail tone; 2 –weakness or paralysis of the hind legs; 3 –strong limb paralysis; 4 –complete paralysis (inability to move); and 5 –death.

**Flow cytometry**

The spleen was isolated from the SJL/J mice of each experimental group. Next, the spleen was transferred to a Petri dish and homogenized to obtain a splenocyte suspension. The isolated splenocytes were resuspended in a DMEM medium, and 1 million cells were centrifuged at 400 g for 10 min, then the precipitate was washed twice with PBS. The cells were added with a solution of the biotinylated MBP peptides (7-TQDENPVVHFFKNIVTPRTPPPS or 12-DAQGTLSKIFKLGGRDSRSGSPMARR) in phosphate buffer containing 1% BSA. The cells were incubated at +4 °C for 40 min, centrifuged (400 g, 10 min), and resuspended in physiological buffer. The cell suspension was supplemented with the anti-B220-APC antibodies (eBioscience, USA) and Streptavidin-Pacific Blue™ conjugate, incubated (+4 °C. 40 min), centrifuged (400 g, 10 min), resuspended in FACS buffer (0.1% BSA, 0.02% sodium azide, 50 μg/mL propidium iodide in phosphate buffer), and analyzed on a FACSDiva flow cytometer (Becton Dickinson, USA).

## RESULTS AND DISCUSSION

**Development of the genetic construct encoding the immunoglobulin constant fragment fused to the MBP peptide**

**Fig. 1:**
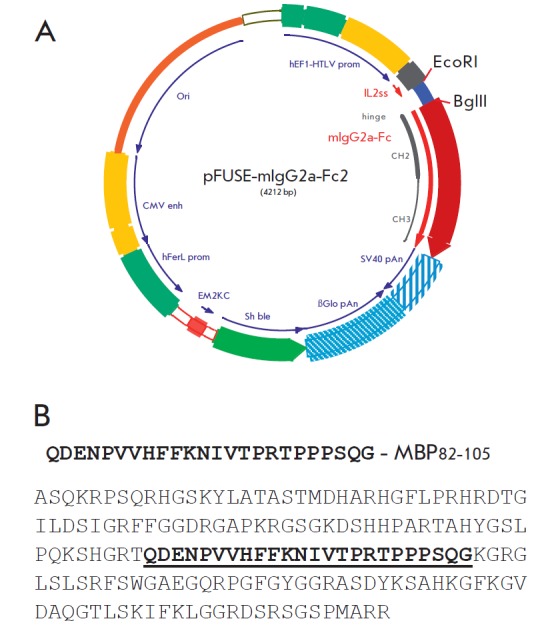
A map of the pFUSE-Fc vector, a plasmid containing cDNA coding for IgG2a Fc. (A) Amino acid sequences of full-size MBP and the MBP_82-105_ fragment (shown in bold) integrated into the pFUSE-Fc vector (B)

To develop highly selective cytotoxic proteins capable of targeted elimination of a population of autoreactive B cells with known specificity, we generated a genetic construct encoding the mouse antibody constant fragment (Fc) fused with the MBP_82–105_ fragment. For this purpose, the commercially available pFUSE plasmid vector containing the gene coding for the constant fragment of mouse IgG2a immunoglobulin (Invivogen) (*Fig. 1*) was used. The nucleotide sequence encoding the MBP_82–105_ (QDENPVVHFFKNIVTPRTPPPSQG) fragment was amplified by PCR with overlapping primers. The resulting DNA fragments were inserted into the pFUSE-mIgG2a plasmid vector at the EcoRI and BgIII restriction sites.

**Fig. 2:**
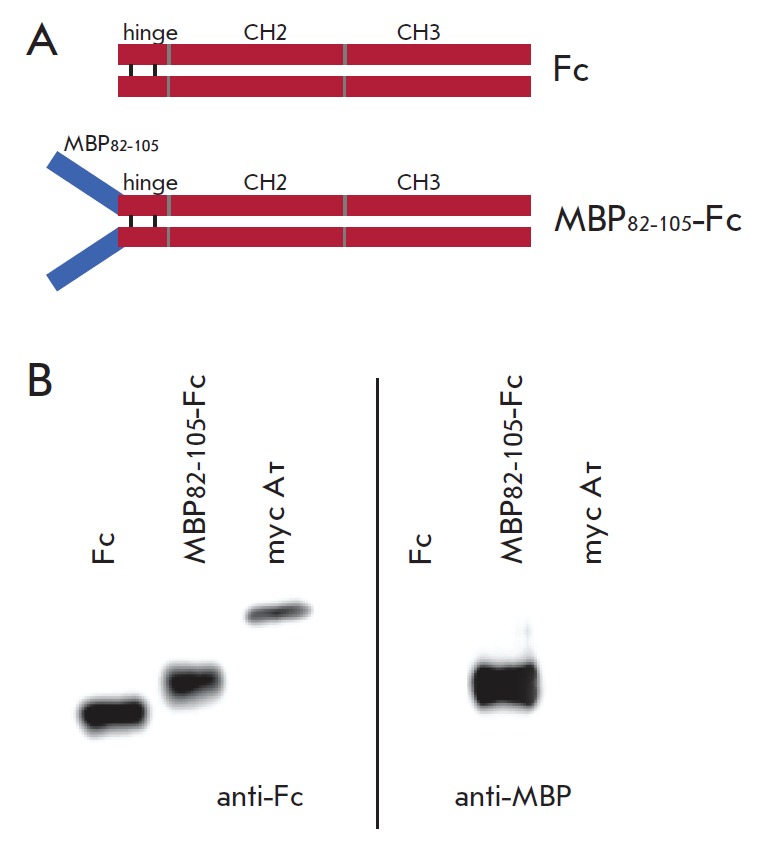
Schematic representation of the developed recombinant Fc and MBP_82-105_-Fc proteins (A). WB analysis of recombinant Fc and MBP_82-105_-Fc using anti-Fc and anti-MBP antibodies

To generate a stable CHO cell line producing the recombinant MBP_82–105_-Fc (pFUSE-MBP_82–105_-Fc) and Fc (pFUSE-Fc) proteins, CHO cell lines were transfected with the appropriate genetic constructs using lipofection. The transfected cells were selected on a medium supplemented with the zeocin antibiotic. The antibioticresistant cells were cloned. The resulting clones were tested for the production of recombinant proteins by ELISA. The selected producer clones were used for preparative production of a protein in 125 cm^2^ flasks for 9 days. The Fc-containing proteins were successively purified from the growth medium by affinity chromatography on a sorbent with the immobilized G protein and on a Superdex200 gel filtration column. According to the electrophoretic analysis, the sample’s homogeneity was beyond 95%. The presence of the MBP fragment in the isolated recombinant proteins was evaluated by Western blotting using the anti-MBP (*Fig. 2B*) and anti- Fc (*Fig. 2A*) antibodies. Hybridization with anti-MBP antibodies confirmed that the fusion MBP_82–05_-Fc protein contained the immunodominant MBP fragment, whereas the control Fc lacked this fragment.

**Depletion of autoreactive B cells in SJL mice with induced EAE**

Experimental autoimmune encephalomyelitis was induced in SJL strain mice to generate a population of autoreactive B cells specific for MBP. For this purpose, animals received two subcutaneous injections of a mouse spinal cord homogenate in a complete Freund’s adjuvant emulsion. Additionally, on the same days, the animals were intravenously injected with a pertussis toxin solution to enhance the development of EAE. Group (1), which included non-immunized mice, was used as a negative control. Further, mice with induced EAE were divided into four experimental groups of 10 animals each: (2) without treatment; (3) animals with a single injection of 200 μg of GA (Teva) (GA group); animals in two groups were intravenously injected twice with Fc (4) and MBP_82–05_-Fc (5) (*Table*).

**Table:** Experimental groups

Group	Number of mice	EAE	Injections	Number of injections	Day of injection	Dose μg/mouse
1	5	-	-	-	-	-
2	10	+	-	-	-	-
3	10	+	GA	1	1	200
4	10	+	Fc	2	5 and 10	50
5	10	+	MBP_82-105_-Fc	2	5 and 10	50

The development of EAE symptoms in all groups was evaluated on a five-grade scale, starting with the seventh day after EAE induction and until the end of the experiment.

**Fig. 3:**
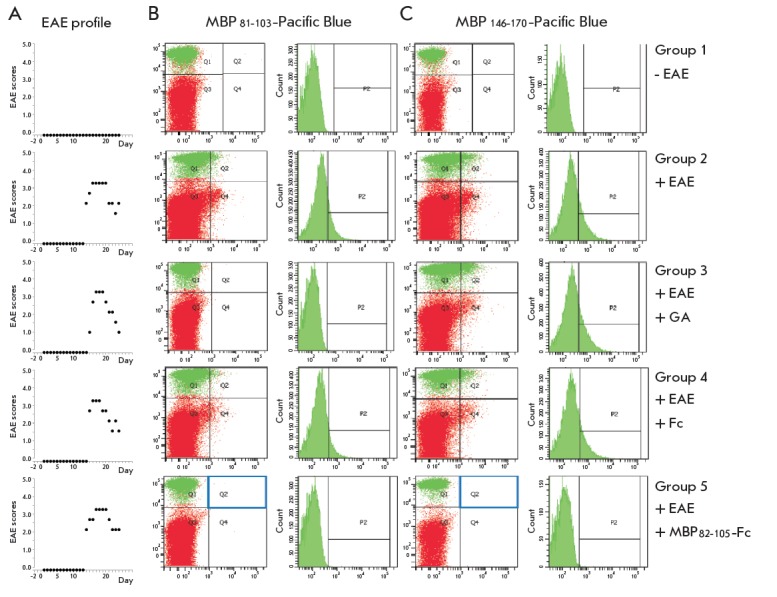
Plots representing the EAE development scores in mice from all experimental groups (A). FC analysis of isolated splenocytes for the presence of B cells specific for the fragments MBP_81-103_ (B) and MBP_146-170_ (C)

EAE symptoms in mice of all groups began to develop on days 14–15 after induction, and the disease peaked on days 17–19. On day 23, SJL mice with identical EAE clinical scores were selected from each experimental group (*Fig. 3A*). The splenocyte cultures derived from these mice were analyzed for B cells specific for the immunodominant and C-terminal MBP fragments. For this purpose, the cells were incubated with the MBP_81–103_ and MBP_146–170_ peptides conjugated with biotin. A conjugate of anti-B220-antibodies with the APC fluorophore (eBioscience) was used to visualize B cells. In turn, the biotinylated MBP peptides bound to the surface BCRs were detected by adding the conjugate of streptavidin with the Pacific Blue™ fluorophore (eBioscience). Samples were analyzed by flow cytometry on the FACSDiva device (BD). As can be seen from *Fig. 3 B,C*, a mouse from the control group lacked a population of B cells specific for MBP, whereas a significant population of B cells specific for both MBP peptides was detected in the culture of splenocytes obtained from an animal from the group without treatment. As expected, intravenous injections of control Fc without MBP peptides did not lead to any change in the population of MBP-reactive B cells. In the case of a single GA injection, the population specific for MBP_81–103_ disappeared in the cell culture but there was a population of B cells with surface BCR specific for the C-terminal MBP_146–170_ fragment. This observation once again confirms that the therapeutic effect of GA is aimed at the population of B cells specific for the immunodominant MBP_81–103_ epitope [24]. Administration of the fusion MBP_81–103_-Fc protein resulted in the complete depletion of B cells specific not only for the MBP_81–103_ fragment, which is a part of the administered immunotoxin, but also for the MBP_146–170_ fragment. Therefore, the profile of the population of B cells specific for MBP in the splenocytes culture of mice subjected to MBP_81–103_-Fc protein therapy coincided with the profile of healthy animals. The obtained results suggest that the MBP_81–103_-Fc immunotoxin, along with the selective depletion of B cells specific for the immunodominant MBP_81–103_ fragment, also inhibits the formation of B cells specific for the MBP_146–170_ fragment.

## CONCLUSIONS

In the last decade, antigen-specific therapy has gained increasing relevance in the development of drugs for the treatment of patients with multiple sclerosis and other autoimmune diseases. By the animal model, we tested one of the most topical current approaches to the treatment of multiple sclerosis – the selective depletion of autoreactive B cells. A highly selective B cell killer protein was generated on the basis of the immunoglobulin constant fragment fused with the immunodominant MBP sequence. Administration of this recombinant immunotoxin to SJL/J mice with induced EAE led to complete elimination of the population of B cells specific for MBP fragments. These findings lead to the conclusion that the suggested concept could be successfully implemented in the development of drugs for targeted therapy of multiple sclerosis and other autoimmune disorders.

## Abbreviations

MBP: myelin basic protein
MS: multiple sclerosis
EAE: experimental autoimmune encephalomyelitis
CFA: complete Freund’s adjuvant
ELISA: enzyme-linked immunosorbent assay
